# Preliminary results on the seroprevalence of *Angiostrongylus vasorum *and co-infection with *Dirofilaria immitis *in shelter dogs from Portugal

**DOI:** 10.1186/1756-3305-7-S1-O26

**Published:** 2014-04-01

**Authors:** AM Alho, M Schnyder, J Meireles, S Belo, P Deplazes, L Madeira de Carvalho

**Affiliations:** 1 Centro de Investigação Interdisciplinar em Sanidade Animal, Faculdade de Medicina Veterinária, Universidade de Lisboa, Portugal; 2 Institute of Parasitology, Vetsuisse Faculty, University of Zurich, Switzerland; 3 Unidade de Parasitologia Médica, Instituto de Higiene e Medicina Tropical, Universidade Nova de Lisboa, Portugal

## 

Angiostrongylosis and dirofilariosis have recently revived the attention of the scientific community due to their emergence in several geographical areas. *Angiostrongylus vasorum *is responsible for verminous pneumonia and also for neurological, cardiovascular and coagulation disorders while *Dirofilaria immitis *is responsible for right side heart failure. Portugal is an endemic country for *D. immitis*. However, prevalence data concerning *A. vasorum *is scarce. Considering that documented cross-reactions between these parasites may lead to incorrect or misfit diagnoses, we aimed to assess the prevalence and potential co-infections of both canine heartworms.

An epidemiological survey was conducted, involving 341 sera collected from shelter dogs housed in three coastal districts of Portugal: Coimbra, Santarém and Setúbal. Sera were tested for circulating *A. vasorum *antigens by a sandwich-ELISA using mono and polyclonal antibodies (sensitivity 95.7%, specificity 94.0%, Schnyder et al., 2011) and for specific antibodies against purified *A. vasorum *adult stage antigen (sensitivity 85.7%, specificity 98.8%, Schucan et al. 2012). In order to detect the presence of *D. immitis *circulating antigens, a commercial kit WITNESS^® ^Dirofilaria (Synbiotics Corp., Europe, sensitivity 100% and specificity 98.3%) based on rapid immunomigration technology was used. Statistical analysis was performed with SPSS program and estimated prevalence was obtained using Agresti-Coull method.

Regarding *A. vasorum*, a total of 1.17% (4/341, 95% Confidence Intervals, CI: 0.3-3.1%) of the animals were positive in both ELISAs, while 1.76% (6/341, CI: 0.7-3.9%) were only antigen-positive and 2.35% (8/341, CI: 1.1-4.6%) only antibody-positive. Positive dogs were from the three surveyed areas. Concerning *D. immitis*, a total of 7.92% of the animals were positive (27/341, CI: 5.5-11.3%), also from the three areas. Out of the 341 sera analysed, one dog was positive for *D. immitis *circulating antigen and *A. vasorum *antibody-positive only.

These results show a slightly higher seroprevalence of angiostrongylosis in Portugal in comparison with seroprevalence found in UK, Italy, Germany and Poland, probably explained by the fact that this study was performed with shelter dogs, usually not under prophylaxis and therefore at a higher risk of infection.

To the authors' knowledge, this is the first study performed in Portugal to assess *A. vasorum *seroprevalence in dogs and *D. immitis *co-infection. We expect that the obtained data will enhance the awareness of veterinary practitioners concerning these diseases and reinforce the importance of a more targeted preventive therapy for heartworms in companion animals in Portugal.

## Funding

PhD research grant SFRH/BD/85427/2012; Project PTDC/SAU-SAP/113523/2009 supported by Fundação para a Ciência e a Tecnologia (FCT), Portugal.

